# Evaluation of Different Dose-Response Models for High Hydrostatic Pressure Inactivation of Microorganisms

**DOI:** 10.3390/foods6090079

**Published:** 2017-09-07

**Authors:** Sencer Buzrul

**Affiliations:** Auditing Department, Tütün ve Alkol Piyasası Düzenleme Kurumu (TAPDK), 06520 Ankara, Turkey; sencer.buzrul@tapdk.gov.tr or sencer.buzrul@gmail.com; Tel.: +90-312-218-0438; Fax: +90-312-220-0605

**Keywords:** high pressure, inactivation kinetics, predictive microbiology, dose-response curves

## Abstract

Modeling of microbial inactivation by high hydrostatic pressure (HHP) requires a plot of the log microbial count or survival ratio versus time data under a constant pressure and temperature. However, at low pressure and temperature values, very long holding times are needed to obtain measurable inactivation. Since the time has a significant effect on the cost of HHP processing it may be reasonable to fix the time at an appropriate value and quantify the inactivation with respect to pressure. Such a plot is called dose-response curve and it may be more beneficial than the traditional inactivation modeling since short holding times with different pressure values can be selected and used for the modeling of HHP inactivation. For this purpose, 49 dose-response curves (with at least 4 log_10_ reduction and ≥5 data points including the atmospheric pressure value (*P* = 0.1 MPa), and with holding time ≤10 min) for HHP inactivation of microorganisms obtained from published studies were fitted with four different models, namely the Discrete model, Shoulder model, Fermi equation, and Weibull model, and the pressure value needed for 5 log_10_ (*P*_5_) inactivation was calculated for all the models above. The Shoulder model and Fermi equation produced exactly the same parameter and *P*_5_ values, while the Discrete model produced similar or sometimes the exact same parameter values as the Fermi equation. The Weibull model produced the worst fit (had the lowest adjusted determination coefficient (R^2^_adj_) and highest mean square error (MSE) values), while the Fermi equation had the best fit (the highest R^2^_adj_ and lowest MSE values). Parameters of the models and also *P*_5_ values of each model can be useful for the further experimental design of HHP processing and also for the comparison of the pressure resistance of different microorganisms. Further experiments can be done to verify the *P*_5_ values at given conditions. The procedure given in this study can also be extended for enzyme inactivation by HHP.

## 1. Introduction

High hydrostatic pressure (HHP) is considered an alternative to traditional preservation techniques for the pasteurization of food products [1,2] and HHP has become a reality in the food industry especially in the past couple of years [3,4,5]. HHP can inactivate microorganisms in food even at room temperature and can minimize the damage to sensitive food components caused by heat [6].

Primary modeling of microbial inactivation by any lethal treatment (heat, pressure, etc.) is simply defined as the reduction in microbial number with time under particular environmental conditions. In other words, in case of HHP inactivation, log microbial count or survival ratio versus time data under a constant pressure (and also temperature) level is obtained and the resulting survival data are fitted with an appropriate model that may be linear or non-linear. Further, secondary and tertiary modeling could be applied to predict microbial inactivation by HHP. Although this type of modeling is useful to predict the microbial inactivation under different pressure conditions, it requires experiments with very long holding times (>30 min), which is not compatible with commercial applications of HHP processing, especially under lower pressure values (<450 MPa).

Evaluation of survival data by HHP in the literature revealed that a long holding time is needed for apparent inactivation, at least for some pressure and temperature combinations. For example, Guan et al. [7] observed about 3 log_10_ and 1.8 reductions of *Salmonella typhimurium* in whole milk after pressurization at 350 MPa, 21 °C for 120 min and at 400 MPa, 21 °C for 30 min, respectively. On the other hand, about 5 log_10_ reduction was observed after 8 min of pressurization at 600 MPa at 21 °C. It should be admitted that 350 MPa for 120 min or 400 MPa for 30 min would never be used to inactivate any microorganisms in any food product commercially because one of the objectives in non-thermal processing is to obtain short processing times [3]. The short processing time (*<*5 or 10 min) is desired since it has a significant effect on the economics of HHP processing [8,9]. According to Mújica-Paz et al. [10], a holding time of 3 min is desirable for the commercial viability of HHP applications. This suggests shorter exposure time at higher pressure values to achieve high microbial inactivation.

The microbial inactivation pattern exposed to a lethal treatment can be described by a dose-response curve [11]. For HHP inactivation, the log survival ratio versus pressure level (dose) at a constant temperature and time should be plotted, and a suitable model should be fitted to describe the data. The use of dose-response modeling for HHP inactivation may be more beneficial than the traditional inactivation modeling since short holding times (*<*5 or 10 min) with different pressure values can be selected and used for modeling purposes. In other words, an experimenter can specify the conditions (time and temperature) under which assessment of the agent’s (pressure) intensity effects is quotable and significant [12]. Therefore, studies on modeling of microbial inactivation by HHP with very long holding time such as ≥60 min can be avoided.

The objective of this study was to demonstrate the usefulness of the dose-response modeling of microbial inactivation by HHP by applying different mathematical models. Furthermore, simulations were performed to show the possible applications of the dose-response modeling for HHP inactivation of microorganisms.

## 2. The Theory

At a constant temperature and time, there is a minimum pressure below which microbial inactivation by HHP will not take place [13]; in other words, at low pressure a delayed effect could be observed and it is expected that a very high pressure can cause almost instantaneous mortality in the exposed microbial population [12]. In many instances the relationship between survival ratio and pressure can be described by the following equations or models.

### 2.1. Discrete Model

If *P* < *P*_min_*S*(*P*) = 1 If *P ≥ P*_min_*S*(*P*) = exp(– *k*(*P* − *P*_min_)) (1)

where *P* is the pressure (in MPa), and *S*(*P*) is the survival ratio i.e., *S*(*P*) = *N*(*P*)/*N*_0_. *N*(*P*) is the number of microorganisms after an exposure of pressure *P* at a constant holding time and temperature and *N*_0_ is the initial number of microorganisms.

In microbial inactivation, the size of the population changes by several orders of magnitude; therefore, expressing survival ratio in log units is appropriate [12]. Moreover, log transformation of microbial population stabilizes the variance [14].

After log transformation, Equation (1) becomes:

(2) $$\begin{array}{c} \mathrm{If}\;p<p_{\min}\;\log_{10}S\left(P\right)=0\\ \mathrm{If}\;p\geq p_{\min}\;\log_{10}S\left(P\right)=-\frac{k}{\ln10}\left(P-P_{\min}\right) \end{array}$$

### 2.2. Fermi Equation

(3) $$\log_{10}S\left(P\right)=\log_{10}\left[\frac{1}{1+\exp\left\{k\left(P-P_{\min}\right)\right\}}\right]$$

or

(4) $$\log_{10}S\left(P\right)=-\log_{10}\left\{1+\exp\left[k\left(P-P_{\min}\right)\right]\right\}$$

According to this equation, when *P* = 0.1 MPa (atmospheric pressure) or when *P* ≪ *P*_min_, log_10_*S*(*P*) ≈ 0, when *P* = *P*_min_, log_10_*S*(*P*) = −0.3. (Note that, if plotted in linear coordinates (if Equation (3) is written without the log transformation), a sigmoidal shape is obtained. In this case, *P*_min_ is the inflection point of *S*(*P*) i.e., *S*(*P*_min_) = 0.5 [15]) When *P* ≫ *P*_min_, log_10_*S*(*P*) ≈ –*k*(*P* − *P*_min_)/ln10. Thus, if HHP inactivation could be described by the Fermi equation, a flat shoulder will appear in the dose-response curve, after which the continuation will look like an almost perfectly straight line [12].

### 2.3. Shoulder Model

Since it may be expected that the dose-response curve has a flat shoulder followed by a linear portion, the following equation, which is commonly used to describe this type of curve [16], was also selected for this study.

(5) $$\log_{10}S(P)=-\frac{kP}{\ln10}+\log_{10}\left(\frac{e^{kP_{\min}}}{1+(e^{kP_{\min}}-1)e^{-kP}}\right)$$

Equation (5) can be rearranged as:

(6) $$\log_{10}S(P)=-\frac{k\left(P-P_{\min}\right)}{\ln10}-\log_{10}\left\{1+\left[(e^{kP_{\min}}-1)e^{-kP}\right]\right\}$$

According to this equation, when *P* = 0.1 MPa or when *P* ≪ *P*_min_, log_10_*S*(*P*) ≈ 0, when *P* = *P*_min_, log_10_*S*(*P*) ≈ –0.3., and when *P* ≫ *P*_min_, log_10_*S*(*P*) ≈ −*k*(*P* − *P*_min_)/ln10.

In all three models above, *P*_min_ (in MPa) is the threshold pressure level that produces a measurable inactivation effect on the microbial population, while *k* (in MPa^−1^) is the slope of the linear portion. Therefore, it is not surprising that the Discrete model, the Fermi equation, and the Shoulder model would produce similar or sometimes identical fits at least for some data.

### 2.4. Weibull Model

Another non-linear and useful model to describe the data with preceding shoulder is the Weibull model [12]:

(7) $$\log_{10}S\left(P\right)=-{\left(\frac{P}{P_{1}}\right)}^{n}$$

where *P*_1_ is the pressure value (in MPa) needed to reduce the initial population, *N*_0_ to *N*_0_/10 and *n* is the shape parameter. The shape parameter, in the case of dose-response curves, is expected to be higher than 1 since the shape of the survival curve is downward and concave—see below.

### 2.5. Calculation of P_5_

The pressure value needed to 5 log_10_ (*P*_5_) could be calculated for all the models above. This calculation is rather straightforward for the Discrete, Fermi, and Weibull models; it may be troublesome for the Shoulder model. Nevertheless, *P*_5_ could be calculated easily with the Solver function of Microsoft Excel^®^ (Microsoft, Washington, DC, USA) for the Shoulder model.

For the Discrete model:

(8) $$P_{5}=\frac{5\ln10}{k}+P_{\min}$$

For the Fermi equation:

(9) $$P_{5}=\frac{\left(10^{5}-1\right)}{k}+P_{\min}$$

For the Weibull model:

(10) P5=51nP1

## 3. The Methodology

### 3.1. Data Selection, Fit of the Models, and Statistical Analysis

A review of published papers reporting microbial inactivation data was performed and dose-response curves with at least 4 log_10_ reduction and ≥ 5 data points (including *P* = 0.1 MPa) were selected. Pressurization with a holding time ≤ 10 min was used since long processing times should be avoided for realistic commercial HHP, as discussed above. A total of 49 dose-response curves were obtained depending upon the microbial inactivation data selection criteria mentioned above; data were taken either from the tables presented or digitized from the figures using a software program (WinDIG 2.5; written by Mr. Dominique Lovy, Geneva, Switzerland), which was also used in other studies [17,18,19].

Data fit of the models and plotting of the results were both performed with SigmaPlot 12.0 (Systat Software Inc., Chicago, IL, USA). The goodness-of-fit of the models was assessed using adjusted determination coefficient (R^2^_adj_) and mean square error (MSE) values.

## 4. Results and Discussion

### 4.1. Goodness-of-Fit of the Models

**Figure 1:**
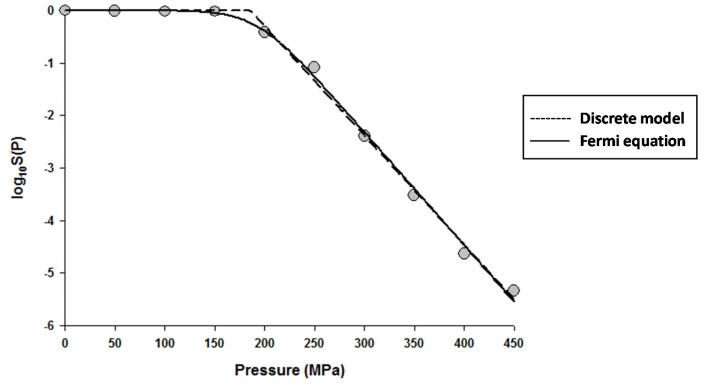
Dose-response curve of *Eschericha coli* in Luria-Bertani broth exposed to high hydrostatic pressure (HHP) at 25 °C for 10 min. Data were fitted with the Discrete model (dashed lines) and the Fermi equation (solid lines). Original data were taken from Moussa et al. [20].

**Figure 2:**
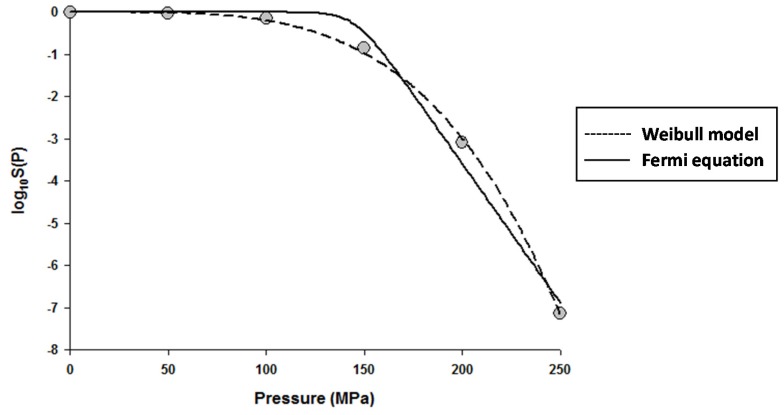
Dose–response curve of *Saccharomyces cerevisiae* in de Man, Rogosa and Sharpe (MRS) broth exposed to high hydrostatic pressure (HHP) at 25 °C for 10 min. Data were fitted with the Weibull model (dashed lines) and the Fermi equation (solid lines). Original data were taken from Donsì et al. [20].

The fitting of the models to the data indicated that the Fermi equation and the Shoulder model produced the exact same fit, parameter estimations, and *P*_5_ values (results not shown). Therefore, only the fit and parameter estimations of the Fermi equation are given throughout this study. Figure 1 shows the fits of the Discrete model and Fermi equation for the inactivation data of *Escherichia coli* in Luria-Bertani (LB) broth. Note that almost identical fits were produced by these two models. Figure 2 shows the fits of the Weibull model and Fermi equation for the inactivation data of *Saccharomyces cerevisiae* in de Man, Rogosa and Sharpe (MRS) broth. Although both models produced reasonable fits, the Weibull model was superior to the Fermi equation for this case.

In general, the three models (Discrete, Fermi, and Weibull) produced good fits for the data, as indicated by their high R^2^_adj_ and low MSE values given in Table 1. The Fermi equation produced the best fit, while the Weibull model gave the worst fit to overall data. Nevertheless, 74% of the Weibull model fits had R^2^_adj_ ≥ 0.95 (80% for Discrete model and 84% for Fermi equation), showing that even the worst-fit model was suitable to describe the dose-response curves for HHP inactivation. The Weibull model also gave slightly higher and much more disperse MSE values than the Discrete model and the Fermi equation (Table 1).

### 4.2. Parameters of the Models

Estimated parameters of the Discrete, Fermi, and Weibull models together with calculated *P*_5_ values are listed in Table 2 for microorganisms in laboratory media and Table 3 for microorganisms in foods. As expected, the Discrete model and the Fermi equation gave similar and sometimes exactly the same parameter estimates. The Weibull model, although it was the worst-fit model among the three models used, gave similar or close *P*_5_ values compared to the Discrete and Fermi models. These tabulated models’ parameters and also the *P*_5_ values of each model can be useful for the further experimental design of HHP processing and also for a comparison of the pressure resistance of different microorganisms.

It should also be noted that data selection was done according to at least 4 log_10_ reduction criteria; however, the pressure value for 5 log_10_ reduction at a constant temperature and time for each microorganism was calculated for each models (Table 2 and Table 3). Thus, extrapolation was inevitable for some data; however, it is highly recommended that the models presented should be used within the interpolation region.

### 4.3. Effects of Substrate on Model Parameters and P_5_ Values

The data presented in Table 2 and Table 3 enable us to investigate the pressure resistance of HHP-treated microorganisms in different substrates/foods. The same strain (ATCC 11775) of *E. coli* in whole milk and LB broth was exposed to HHP by both Buzrul et al. [39,40] and Wang et al. [24], respectively. Processing conditions (temperature and time) were the same, but compression and decompression were faster in the study of Wang et al. [24]. These studies could be used to differentiate the HHP inactivation of microorganisms in foods and laboratory media. It is known that the bacteria in milk are more pressure-resistant than the bacteria in a buffer system [47]. This was also supported by the data given in Table 2 and Table 3. Based on the Fermi equation, *P*_min_ = 278.6 ± 11.5 MPa for *E. coli* in LB broth (Table 2) while it was *P*_min_ = 380.1 ± 15.7 MPa in whole milk (Table 3). Therefore, the pressure needed to obtain a measurable HHP inactivation (20–22 °C, 10 min) of *E. coli* in milk was about 100 MPa higher than in broth. Similarly, the *P*_5_ value for milk (582.1 MPa) was higher than for broth (426.2 MPa). Moreover, inactivation increased more as the pressure increased in broth than in milk since the slope, i.e., *k* value, was higher in broth (0.078 ± 0.008 MPa^−1^) than in milk (0.057 ± 0.006 MPa^−1^).

Another example of the effect of substrate is the study of Solomon and Hoover [21], in which *Campylobacter jejuni* (ATCC 35921) was exposed to HHP in Bolton broth and whole milk. Similar results for the Fermi equation were also obtained in the study by Solomon and Hoover [21]. The same goes for both *E. coli* and *C. jejuni* in terms of the *P*_1_ of the Weibull model—see Table 2 and Table 3.

### 4.4. Effect of Holding Time, Temperature, and Compression and Decompression Rates on Model Parameters and P_5_ Values

Although it was not easy to make definitive conclusions due to the limited data at hand, it may be expected that as the holding time and temperature increase, *P*_min_ decreases and *k* increases for the Discrete and Fermi models and *P*_1_ decreases for the Weibull model. In traditional modeling, where time is a variable, the shape parameter of the Weibull model is independent or only a weak function of temperature [48] and pressure [19]. In the case of dose-response modeling, it was generally observed that as the time/temperature increased the shape parameter decreased—see the data of Doğan and Erkmen [22] and Donsì et al. [32] in Table 2. However, just the opposite was also observed—see the data of Doona et al. [38] in Table 3.

The effect of holding time can be clearly observed in Table 2 for the data of Doğan and Erkmen [22], in which *E. coli* was exposed to different pressure values at 25 °C for 3, 5, and 10 min. The decrease of *P*_min_ (for the Discrete and Fermi models) and *P*_1_ (the Weibull model) could be observed for the increase in time. The same can be said about temperature for *P*_min_ and *P*_1_ values (Table 1 and Table 2); however, there were some exceptions for *k*: decreases in *k* were observed if the time (or temperature) of HHP treatment increased. Some examples of such a situation are given in the studies of Wang et al. [33] (Table 2) and Carreño et al. [42] (Table 3).

It is known that rates of compression and decompression may have an impact on microbial inactivation [49]; however, the effect of the rates on models’ parameters cannot be shown due to a lack of data. Nevertheless, the rates or times of compression and decompression are given in Table 2 and Table 3.

### 4.5. Possible Applications of the Fermi Equation

**Figure 3:**
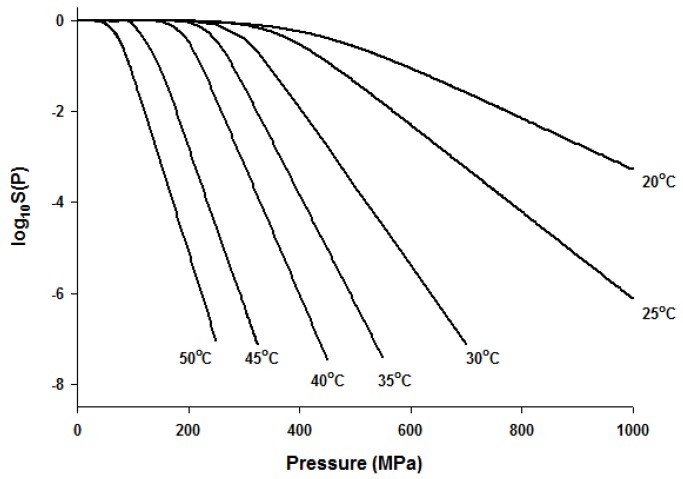
Simulated dose-response curves with the Fermi equation for microbial inactivation by HHP at different temperatures.

**Figure 4:**
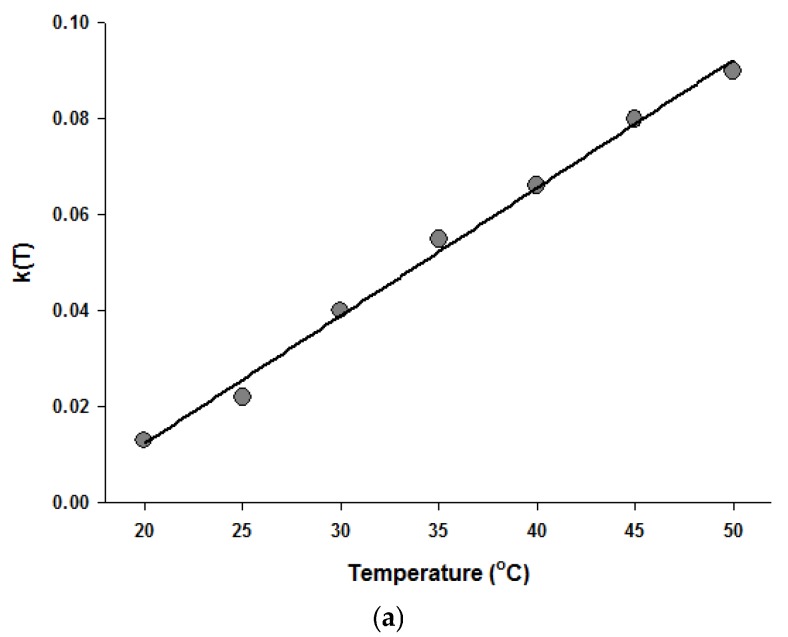

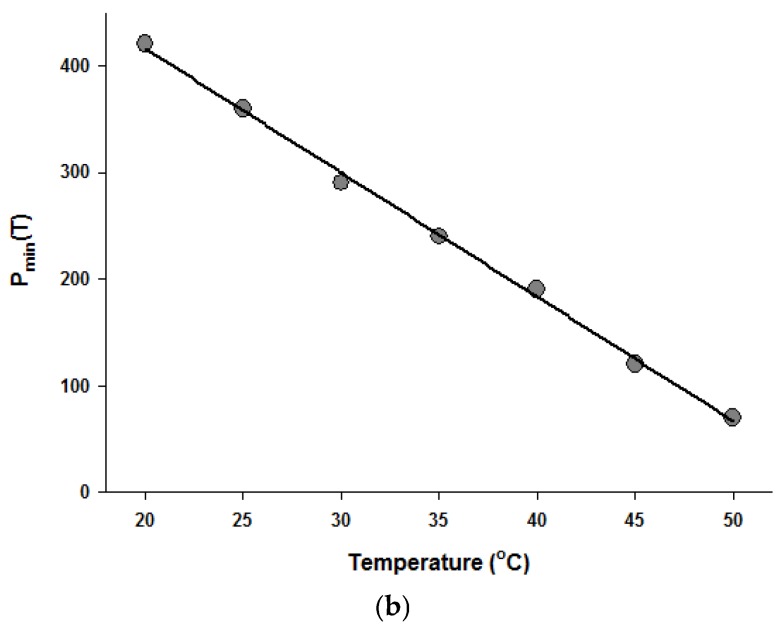
Temperature dependence of the parameters of the Fermi equation: *k* (**a**) and *P*_min_ (**b**).

Since the Fermi equation was the best-fit model among the models tested, some possible applications of the Fermi equation were simulated. The usefulness of the Fermi equation was also demonstrated in the modeling of thermal inactivation [50] and pulse electrical field inactivation [51]. Figure 3 shows the simulated dose-response curves with the Fermi equation at different temperatures at a constant holding time. Figure 4 shows the effect of temperature on the parameters of the Fermi equation. Note that any empirical model could be used to describe the temperature dependence of *k*(*T*) and *P*_min_(*T*). Moreover, this type of modeling can also be used to define the time dependency of *k*(*t*) and *P*_min_(*t*) or the temperature and time dependency of *k*(*t*,*T*) and *P*_min_(*t*,*T*). As mentioned before, dose–response modeling of microbial inactivation by HHP could be more beneficial than the traditional inactivation modeling, where long holding times are needed for high inactivation values, especially at low pressure values.

Selection of appropriate holding time(s) (3 or 5 min) can be the first step in the experimental design of dose-response modeling of HHP inactivation. Then a trial with a pressure value (200 MPa) at a constant temperature (20 °C) could be made to quantify the inactivation. If no inactivation is observed, it may be assumed that pressure values lower than the selected pressure level (200 MPa) would also have no effect on microbial inactivation. Then, pressure can be increased incrementally (50 MPa) to observe the measurable inactivation and total inactivation (8 log_10_) if possible. The procedure could be repeated each time with a new temperature value.

## 5. Conclusions and Future Outlook

The processing costs of HHP treatment are related to the holding times at the target pressure [52]; this is why shorter treatment times would be economically beneficial. Dose-response modeling can allow us to use short treatment times (≤ 10 min). The models used in this study were all suitable to describe the HHP inactivation; however, the Fermi equation was the best-fit model with two interpretable parameters. The pressure value needed for measurable inactivation (*P*_min_ in MPa), which corresponds to 0.3 log_10_ inactivation or inactivation of half of the initial population, i.e., *N*_0_ to *N*_0_/2, and slope (*k* in MPa^−1^) of the inactivation could be estimated.

In fact, the procedure given in this study can also be extended for enzyme inactivation by HHP. Moreover, inactivation due to compression and decompression should be quantified and dose-response modeling should be done with inactivation at the constant pressure values. The effect of temperature during HHP treatment and injury of microorganisms should also be taken into account. Further experiments can be done to verify the *P*_5_ values at the given conditions.

## Figures and Tables

**Table 1:** Adjusted coefficient determination (R^2^_adj_) and mean square error (MSE) values for the Discrete, Fermi, and Weibull models applied to 49 dose-response curves obtained from the literature.

	R^2^_adj_			MSE		
	Discrete	Fermi	Weibull	Discrete	Fermi	Weibull
Mean	0.970	0.973	0.967	0.224	0.201	0.295
Standard deviation	0.025	0.022	0.031	0.145	0.137	0.317
Min	0.900	0.917	0.872	0.009	0.007	0.005
Max	0.999	0.999	0.999	0.640	0.638	1.36

**Table 2:** Parameters and *P*_5_ values of the Discrete, Fermi, and Weibull models applied to microorganisms exposed to high hydrostatic pressure (HHP) in laboratory media.

Microorganism	Strain	Substrate	CR/CT ^a^	DR/DT ^b^	Process Conditions ^c^	Discrete	Fermi	Weibull	Reference
*Campylobacter jejuni*	ATCC 35921	Bolton broth	ND ^d^	ND	25 °C, 10 min	*k* = 0.307 ± 0.021 MPa^−1^	*k* = 0.309 ± 0.021 MPa^−1^	*n* = 8.6 ± 0.4	Solomon & Hoover [21]
						*P*_min_ = 241.0 ± 2.7 MPa	*P*_min_ = 241.3 ± 2.8 MPa	*P*_1_ = 235.0 ± 2.7 MPa	
						*P*_5_ = 278.5 MPa	*P*_5_ = 278.6 MPa	*P*_5_ = 283.4 MPa	
*Escherichia coli*	KUEN 1504	Brain heart infusion broth	100 MPa·s^−1^	200 MPa·s^−1^	25 °C, 3 min	*k* = 0.022 ± 0.001 MPa^−1^	*k* = 0.023 ± 0.001 MPa^−1^	*n* = 1.7 ± 0.2	Doğan & Erkmen [22]
						*P*_min_ = 175.9 ± 10.9 MPa	*P*_min_ = 190.8 ± 12.5 MPa	*P*_1_ = 262.1 ± 21.4 MPa	
						*P*_5_ = 699.2 MPa	*P*_5_ = 691.4 MPa	*P*_5_ = 675.5 MPa	
					25 °C, 5 min	*k* = 0.031 ± 0.001 MPa^−1^	*k* = 0.032 ± 0.002 MPa^−1^	*n* = 1.6 ± 0.2	
						*P*_min_ = 165.8 ± 15.1 MPa	*P*_min_ = 172.8 ± 16.6 MPa	*P*_1_ = 202.2 ± 22.0 MPa	
						*P*_5_ = 537.2 MPa	*P*_5_ = 532.6 MPa	*P*_5_ = 552.9 MPa	
					25 °C, 10 min	*k* = 0.040 ± 0.003 MPa^−1^	*k* = 0.040 ± 0.003 MPa^−1^	*n* = 1.5 ± 0.2	
						*P*_min_ = 138.6 ± 20.5 MPa	*P*_min_ = 139.1 ± 21.8 MPa	*P*_1_ = 147.2 ± 27.1 MPa	
						*P*_5_ = 426.4 MPa	*P*_5_ = 426.9 MPa	*P*_5_ = 430.4 MPa	
*E. coli*	ATCC 25922	Phosphate-buffered saline	ND	ND	25 °C, 5 min	*k* = 0.113 ± 0.010 MPa^−1^	*k* = 0.113 ± 0.010 MPa^−1^	*n* = 2.5 ± 0.4	Yamamoto et al. [23]
						*P*_min_ = 134.1 ± 9.8 MPa	*P*_min_ = 134.4 ± 9.9 MPa	*P*_1_ = 127.8 ± 14.7 MPa	
						*P*_5_ = 236.0 MPa	*P*_5_ = 236.3 MPa	*P*_5_ = 243.3 MPa	
					25 °C, 10 min	*k* = 0.116 ± 0.011 MPa^−1^	*k* = 0.116 ± 0.012 MPa^−1^	*n* = 2.4 ± 0.5	
						*P*_min_ = 131.6 ± 9.5 MPa	*P*_min_ = 131.3 ± 10.2 MPa	*P*_1_ = 122.2 ± 19.1 MPa	
						*P*_5_ = 230.9 MPa	*P*_5_ = 230.6 MPa	*P*_5_ = 239.0 MPa	
*E. coli*	K12TG1	Luria-Bertani broth	3 min	3 min	25 °C, 10 min	*k* = 0.048 ± 0.002 MPa^−1^	*k* = 0.050 ± 0.002 MPa^−1^	*n* = 2.4 ± 0.2	Moussa et al. [20]
						*P*_min_ = 185.7 ± 5.2 MPa	*P*_min_ = 192.3 ± 5.6 MPa	*P*_1_ = 218.9 ± 13.4 MPa	
						*P*_5_ = 425.6 MPa	*P*_5_ = 422.6 MPa	*P*_5_ = 428.0 MPa	
*E. coli*	ATCC 11775	Luria-Bertani broth	10 s	10 s	20 °C, 10 min	*k* = 0.062 ± 0.007 MPa^−1^	*k* = 0.078 ± 0.008 MPa^−1^	*n* = 4.3 ± 0.1	Wang et al. [24]
						*P*_min_ = 249.9 ± 14.2 MPa	*P*_min_ = 278.6 ± 11.5 MPa	*P*_1_ = 293.4 ± 2.6 MPa	
						*P*_5_ = 435.6 MPa	*P*_5_ = 426.2 MPa	*P*_5_ = 426.6 MPa	
					30 °C, 10 min	*k* = 0.050 ± 0.006 MPa^−1^	*k* = 0.051 ± 0.006MPa^−1^	*n* = 2.6 ± 0.2	
						*P*_min_ = 168.6 ± 22.3 MPa	*P*_min_ = 172.8 ± 22.3 MPa	*P*_1_ = 214.2 ± 11.5 MPa	
						*P*_5_ = 398.9 MPa	*P*_5_ = 398.5 MPa	*P*_5_ = 397.8 MPa	
*E. coli*	MG1655	Luria-Bertani broth	50 MPa·s^−1^	<1 s	25 °C, 10 min	*k* = 0.054 ± 0.002 MPa^−1^	*k* = 0.054 ± 0.002 MPa^−1^	*n* = 2.6 ± 0.2	Griffin et al. [25]
						*P*_min_ = 182.1 ± 5.3 MPa	*P*_min_ = 184.1 ± 5.0 MPa	*P*_1_ = 211.4 ± 9.0 MPa	
						*P*_5_ = 395.3 MPa	*P*_5_ = 397.3 MPa	*P*_5_ = 392.6 MPa	
*Lactobacillus plantarum*	103151T	MRS broth	1.5 MPa·s^−1^	1.5 MPa·s^−1^	25 °C, 10 min	*k* = 0.182 ± 0.010 MPa^−1^	*k* = 0.214 ± 0.009 MPa^−1^	*n* = 5.5 ± 0.6	Perrier-Cornet et al. [26]
						*P*_min_ = 199.6 ± 3.6 MPa	*P*_min_ = 212.2 ± 2.7 MPa	*P*_1_ = 203.9 ± 8.3 MPa	
						*P*_5_ = 262.9 MPa	*P*_5_ = 266.0 MPa	*P*_5_ = 273.2 MPa	
*L. viridescens*	IFO 3949	MRS broth	1 min	≈2 s	25 °C, 5 min	*k* = 0.065 ± 0.013 MPa^−1^	*k* = 0.065 ± 0.013 MPa^−1^	*n* =3.0 ± 0.5	Park et al. [27]
						*P*_min_ = 319.6 ± 39.6 MPa	*P*_min_ = 319.7 ± 37.7 MPa	*P*_1_ = 298.2 ± 55.4 MPa	
						*P*_5_ = 496.7 MPa	*P*_5_ = 496.8 MPa	*P*_5_ = 509.9 MPa	
*Listeria monocytogenes*	Poultry isolate	Buffered saline	≈ 2 min	1 min	AT ^e^, 5 min	*k* = 0.101 ± 0.004 MPa^−1^	*k* = 0.104 ± 0.006 MPa^−1^	*n* = 4.5 ± 0.7	Simpson & Gilmour [28]
						*P*_min_ = 296.3 ± 3.7 MPa	*P*_min_ = 299.0 ± 5.8 MPa	*P*_1_ = 291.2 ± 18.0 MPa	
						*P*_5_ = 410.3 MPa	*P*_5_ = 409.7 MPa	*P*_5_ = 416.4 MPa	
					AT, 10 min	*k* = 0.112 ± 0.012 MPa^−1^	*k* = 0.112 ± 0.013MPa^−1^	*n* =3.5 ± 0.8	
						*P*_min_ = 279.8 ± 11.5 MPa	*P*_min_ = 279.4 ± 12.6 MPa	*P*_1_ = 246.4 ± 31.2 MPa	
						*P*_5_ = 382.6 MPa	*P*_5_ = 382.2 MPa	*P*_5_ = 390.3 MPa	
	Scott A					*k* = 0.080 ± 0.011 MPa^−1^	*k* = 0.081 ± 0.011 MPa^−1^	*n* = 5.3 ± 0.9	
						*P*_min_ = 313.7 ± 12.8 MPa	*P*_min_ = 315.5 ± 12.3 MPa	*P*_1_ = 333.2 ± 14.6 MPa	
						*P*_5_ = 457.6 MPa	*P*_5_ = 457.6 MPa	*P*_5_ = 451.4 MPa	
*L. monocytogenes*	Scott A	Citrate buffer	ND	ND	20 °C, 10 min	*k* = 0.105 ± 0.028 MPa^−1^	*k* = 0.105 ± 0.028 MPa^−1^	*n* = 3.9 ± 0.9	Tholozan et al. [29]
						*P*_min_ = 227.2 ± 31.8 MPa	*P*_min_ = 227.4 ± 31.7 MPa	*P*_1_ = 231.8 ± 25.7 MPa	
						*P*_5_ = 336.9 MPa	*P*_5_ = 337.1 MPa	*P*_5_ = 350.2 MPa	
		Phosphate buffer				*k* = 0.061 ± 0.008 MPa^−1^	*k* = 0.061 ± 0.008 MPa^−1^	*n* = 2.4 ± 0.5	
						*P*_min_ = 271.6 ± 28.2 MPa	*P*_min_ = 271.6 ± 28.4 MPa	*P*_1_ = 245.9 ± 45.0 MPa	
						*P*_5_ = 460.3 MPa	*P*_5_ = 460.3 MPa	*P*_5_ = 480.8 MPa	
*L. monocytogenes*	4a KUEN 136	Brain heart infusion broth	100 MPa·s^−1^	200 MPa·s^−1^	25 °C, 5 min	*k* = 0.024 ± 0.002 MPa^−1^	*k* = 0.024 ± 0.003 MPa^−1^	*n* = 1.3 ± 0.2	Erkmen & Dogan [30]
						*P*_min_ = 119.0 ± 37.7 MPa	*P*_min_ = 122.5 ± 40.0 MPa	*P*_1_ = 178.5 ± 33.7 MPa	
						*P*_5_ = 598.7 MPa	*P*_5_ = 602.2 MPa	*P*_5_ = 615.6 MPa	
					25 °C, 10 min	*k* = 0.031 ± 0.003 MPa^−1^	*k* = 0.031 ± 0.003 MPa^−1^	*n* = 1.2 ± 0.2	
						*P*_min_ = 91.2 ± 31.7 MPa	*P*_min_ = 91.6 ± 32.2 MPa	*P*_1_ = 124.1 ± 23.7 MPa	
						*P*_5_ = 462.6 MPa	*P*_5_ = 463.0 MPa	*P*_5_ = 474.5 MPa	
*L. monocytogenes*	ATCC 19117	1% buffered peptone water	5 MPa·s^−1^	<10 s	25 °C, 10 min	*k* = 0.069 ± 0.010 MPa^−1^	*k* = 0.071 ± 0.009MPa^−1^	*n* = 2.9 ± 0.8	Koseki & Yamamoto [31]
						*P*_min_ = 304.9 ± 30.2 MPa	*P*_min_ = 311.3 ± 26.8 MPa	*P*_1_ = 284.9 ± 53.3 MPa	
						*P*_5_ = 471.8 MPa	*P*_5_ = 473.5 MPa	*P*_5_ = 496.3 MPa	
		5% buffered peptone water				*k* = 0.084 ± 0.010 MPa^−1^	*k* = 0.112 ± 0.009MPa^−1^	*n* = 5.9 ± 0.4	
						*P*_min_ = 398.5 ± 15.3 MPa	*P*_min_ = 441.9 ± 9.5 MPa	*P*_1_ = 423.5 ± 8.8 MPa	
						*P*_5_ = 535.6 MPa	*P*_5_ = 544.7 MPa	*P*_5_ = 556.3 MPa	
*Saccharomyces cerevisiae*	ND	MRS broth	ND	≈2 s	25 °C, 10 min	*k* = 0.145 ± 0.012 MPa^−1^	*k* = 0.152 ± 0.014 MPa^−1^	*n* = 3.9 ± 0.2	Donsì et al. [32]
						*P*_min_ = 141.3 ± 6.1 MPa	*P*_min_ = 145.6 ± 7.0 MPa	*P*_1_ = 151.4 ± 1.9 MPa	
						*P*_5_ = 220.7 MPa	*P*_5_ = 221.3 MPa	*P*_5_ = 228.7 MPa	
					45 °C, 3 min	*k* = 0.140 ± 0.012 MPa^−1^	*k* = 0.144 ± 0.023 MPa^−1^	*n* =3.8 ± 0.2	
						*P*_min_ = 109.6 ± 9.3 MPa	*P*_min_ = 111.7 ± 9.5 MPa	*P*_1_ = 126.4 ± 3.9 MPa	
						*P*_5_ = 191.8 MPa	*P*_5_ = 191.7 MPa	*P*_5_ = 193.1 MPa	
					45 °C, 6 min	*k* = 0.140 ± 0.01 MPa^−1^	*k* = 0.146 ± 0.014 MPa^−1^	*n* =3.0 ± 0.1	
						*P*_min_ = 93.1 ± 5.9 MPa	*P*_min_ = 96.2 ± 6.2 MPa	*P*_1_ = 105.1 ± 2.3 MPa	
						*P*_5_ = 175.3 MPa	*P*_5_ = 175.1 MPa	*P*_5_ = 179.7 MPa	
					45 °C, 10 min	*k* = 0.141 ± 0.011 MPa^−1^	*k* = 0.144 ± 0.012 MPa^−1^	*n* = 2.8 ± 0.09	
						*P*_min_ = 89.9 ± 5.2 MPa	*P*_min_ = 91.7 ± 5.3 MPa	*P*_1_ = 98.9 ± 2.1 MPa	
						*P*_5_ = 171.6 MPa	*P*_5_ = 171.7 MPa	*P*_5_ = 175.7 MPa	
*S. cerevisiae*	CBS 1171	Malt Wickerham medium	1.5 MPa·s^−1^	1.5 MPa·s^−1^	25 °C, 10 min	*k* = 0.165 ± 0.013 MPa^−1^	*k* = 0.166 ± 0.014 MPa^−1^	*n* = 4.1 ± 0.2	Perrier-Cornet et al. [26]
						*P*_min_ = 184.1 ± 6.3 MPa	*P*_min_ = 184.8 ± 6.5 MPa	*P*_1_ = 177.7 ± 3.4 MPa	
						*P*_5_ = 253.9 MPa	*P*_5_ = 254.2 MPa	*P*_5_ = 263.1 MPa	
*Salmonella enterica*	BCRC 12947	Nutrient broth	45 MPa·s^−1^	<10 s	25 °C, 5 min	*k* = 0.122 ± 0.028 MPa^−1^	*k* = 0.122 ± 0.027MPa^−1^	*n* = 2.9 ± 0.3	Wang et al. [33]
						*P*_min_ = 175.5 ± 2.6 MPa	*P*_min_ = 175.6 ± 2.5 MPa	*P*_1_ = 162.0 ± 11.4 MPa	
						*P*_5_ = 269.9 MPa	*P*_5_ = 270.0 MPa	*P*_5_ = 282.2 MPa	
					25 °C, 10 min	*k* = 0.086 ± 0.006 MPa^−1^	*k* = 0.090 ± 0.006MPa^−1^	*n* = 1.8 ± 0.2	
						*P*_min_ = 94.1 ± 10.0 MPa	*P*_min_ = 102.5 ± 10.8 MPa	*P*_1_ = 97.8 ± 12.1 MPa	
						*P*_5_ = 228.0 MPa	*P*_5_ = 230.4 MPa	*P*_5_ = 239.1 MPa	
*S. enteritidis*	FDA	Trypticase soy broth + yeast extract	6.7 MPa·s^−1^	ND	25 °C, 10 min	*k* = 0.073 ± 0.006 MPa^−1^	*k* = 0.074 ± 0.007 MPa^−1^	*n* = 2.9 ± 0.4	Lee & Kaletunç [34]
						*P*_min_ = 235.7 ± 14.2 MPa	*P*_min_ = 237.3 ± 16.2 MPa	*P*_1_ = 235.0 ± 20.7 MPa	
						*P*_5_ = 393.4 MPa	*P*_5_ = 392.9 MPa	*P*_5_ = 409.4 MPa	
*S. typhimurium*	ATCC 13311	Citrate buffer	ND	ND	20 °C, 10 min	*k* = 0.110 ± 0.005MPa^−1^	*k* = 0.111 ± 0.005MPa^−1^	*n* =3.2 ± 0.3	Tholozan et al. [29]
						*P*_min_ = 177.5 ± 5.6 MPa	*P*_min_ = 178.6 ± 5.7 MPa	*P*_1_ = 180.3 ± 11.7 MPa	
						*P*_5_ = 282.2 MPa	*P*_5_ = 282.3 MPa	*P*_5_ = 298.1 MPa	
		Phosphate buffer				*k* = 0.157 ± 0.013MPa^−1^	*k* = 0.156 ± 0.012MPa^−1^	*n* = 4.9 ± 0.4	
						*P*_min_ = 276.4 ± 7.5 MPa	*P*_min_ = 275.9 ± 7.5 MPa	*P*_1_ = 258.0 ± 7.7 MPa	
						*P*_5_ = 349.7 MPa	*P*_5_ = 349.7 MPa	*P*_5_ = 358.3 MPa	
*S. typhimurium*	KUEN 1357	Tryptone soy broth	100 MPa·s^−1^	200 MPa·s^−1^	25 °C, 10 min	*k* = 0.057 ± 0.005 MPa^−1^	*k* = 0.057 ± 0.005 MPa^−1^	*n* = 2.1 ± 0.4	Erkmen [35]
						*P*_min_ = 163.3 ± 12.7 MPa	*P*_min_ = 163.8 ± 14.1 MPa	*P*_1_ = 171.8 ± 22.9 MPa	
						*P*_5_ = 365.3 MPa	*P*_5_ = 365.8 MPa	*P*_5_ = 369.7 MPa	
*Vibrio parahaemolyticus*	BCRC 10806	Tryptic soy broth	45 MPa·s^−1^	<10 s	25 °C, 5 min	*k* = 0.185 ± 0.013 MPa^−1^	*k* = 0.190 ± 0.014MPa^−1^	*n* = 5.7 ± 0.3	Wang et al. [36]
						*P*_min_ = 241.8 ± 5.0 MPa	*P*_min_ = 244.2 ± 5.7 MPa	*P*_1_ = 237.2 ± 4.6 MPa	
						*P*_5_ = 304.0 MPa	*P*_5_ = 304.8 MPa	*P*_5_ = 314.6 MPa	
					25 °C, 10 min	*k* = 0.132 ± 0.012 MPa^−1^	*k* = 0.162 ± 0.014MPa^−1^	*n* = 3.9 ± 0.4	
						*P*_min_ = 196.1 ± 9.1 MPa	*P*_min_ = 218.8 ± 8.1 MPa	*P*_1_ = 196.6 ± 11.2 MPa	
						*P*_5_ = 283.3 MPa	*P*_5_ = 289.9 MPa	*P*_5_ = 297.0 MPa	

^a^ CR, Compression rate; CT, Compression time. ^b^ DR, Decompression rate; DT, Decompression time. ^c^ The temperature given is either the initial or the process temperature of the treatment. ^d^ ND, Not determined. ^e^ AT, Ambient temperature.

**Table 3:** Parameters and *P*_5_ values of the Discrete, Fermi, and Weibull models applied to microorganisms exposed to high hydrostatic pressure (HHP) in foods.

Microorganism	Strain	Substrate	CR/CT ^a^	DR/DT ^b^	Process Conditions ^c^	Discrete	Fermi	Weibull	Reference
*Campylobacter jejuni*	ATCC 35921	Whole milk	ND ^d^	ND	25 °C, 10 min	*k* = 0.203 ± 0.025 MPa^−1^	*k* = 0.217 ± 0.030 MPa^−1^	*n* = 9.0 ± 0.3	Solomon & Hoover [21]
						*p*_min_ = 293.6 ± 6.5 MPa	*p*_min_ = 298.0 ± 7.3 MPa	*p*_1_ = 298.3 ± 2.1 MPa	
						*p*_5_ = 350.3 MPa	*p*_5_ = 351.1 MPa	*p*_5_ = 356.7 MPa	
*E. coli*	405 CECT	Liquid whole egg	3–4 min	90–120 s	50 °C, 5 min	*k* = 0.061 ± 0.012 MPa^−1^	*k* = 0.074 ± 0.016MPa^−1^	*n* = 5.3 ± 0.4	Ponce et al. [37]
						*p*_min_ = 286.9 ± 21.0 MPa	*p*_min_ = 309.7 ± 20.9 MPa	*p*_1_ = 334.3 ± 7.3 MPa	
						*p*_5_ = 475.6 MPa	*p*_5_ = 465.3 MPa	*p*_5_ = 452.9 MPa	
*E. coli*	ATCC 11229	Whey protein solution	ND	ND	50 °C, 5 min	*k* = 0.095 ± 0.003 MPa^−1^	*k* = 0.098 ± 0.003 MPa^−1^	*n* = 5.0 ± 0.8	Doona et al. [38]
						*p*_min_ = 229.1 ± 2.7 MPa	*p*_min_ = 231.8 ± 2.8 MPa	*p*_1_ = 250.3 ± 12.4 MPa	
						*p*_5_ = 350.3 MPa	*p*_5_ = 349.3 MPa	*p*_5_ = 345.4 MPa	
					50 °C, 10 min	*k* = 0.137 ± 0.006 MPa^−1^	*k* = 0.138 ± 0.006 MPa^−1^	*n* = 5.6 ± 1.0	
						*p*_min_ = 222.0 ± 2.6 MPa	*p*_min_ = 222.4 ± 2.7 MPa	*p*_1_ = 229.4 ± 11.9 MPa	
						*p*_5_ = 306.0 MPa	*p*_5_ = 305.8 MPa	*p*_5_ = 305.8 MPa	
*E. coli*	ATCC 11775	Whole milk	5 MPa·s^−1^	5 MPa·s^−1^	22 °C, 10 min	*k* = 0.057 ± 0.006 MPa^−1^	*k* = 0.057 ± 0.006 MPa^−1^	*n* = 4.4 ± 0.2	Buzrul et al. [39,40]
						*p*_min_ = 378.8 ± 14.9 MPa	*p*_min_ = 380.1 ± 15.7 MPa	*p*_1_ = 402.1 ± 4.8 MPa	
						*p*_5_ = 580.8 MPa	*p*_5_ = 582.1 MPa	*p*_5_ = 579.7 MPa	
*E. coli, O157:H7*	Cocktail of 5 strains	puree	22 MPa·s^−1^	<10 s	21 °C, 2 min	*k* = 0.050 ± 0.009 MPa^−1^	*k* = 0.056 ± 0.010 MPa^−1^	*n* = 3.3 ± 0.3	Huang et al. [41]
						*p*_min_ = 183.3 ± 24.2 MPa	*p*_min_ = 201.8 ± 24.6 MPa	*p*_1_ = 240.4 ± 10.6 MPa	
						*p*_5_ = 413.6 MPa	*p*_5_ = 407.4 MPa	*p*_5_ = 391.5 MPa	
*Lactobacillus plantarum*	CECT 220	Mandarin juice	90 s	15 s	30 °C, 1 min	*k* = 0.106 ± 0.018 MPa^−1^	*k* = 0.099 ± 0.015 MPa^−1^	*n* = 6.8 ± 0.5	Carreño et al. [42]
						*p*_min_ = 307.6 ± 10.6 MPa	*p*_min_ = 302.8 ± 9.8 MPa	*p*_1_ = 321.1 ± 4.7 MPa	
						*p*_5_ = 416.2 MPa	*p*_5_ = 419.1 MPa	*p*_5_ = 406.9 MPa	
					45 °C, 1 min	*k* = 0.081 ± 0.007 MPa^−1^	*k* = 0.081 ± 0.007 MPa^−1^	*n* = 3.4 ± 0.3	
						*p*_min_ = 225.5 ± 10.2 MPa	*p*_min_ = 226.7 ± 10.3 MPa	*p*_1_ = 231.5 ± 9.7 MPa	
						*p*_5_ = 367.6 MPa	*p*_5_ = 368.8 MPa	*p*_5_ = 371.7 MPa	
*Listeria innocua*	ATCC 33090	Whole milk	5 MPa·s^−1^	5 MPa·s^−1^	22 °C, 10 min	*k* = 0.086 ± 0.013 MPa^−1^	*k* = 0.086 ± 0.013 MPa^−1^	*n* = 5.6 ± 0.4	Buzrul et al. [39,40]
						*p*_min_ = 426.5 ± 17.1 MPa	*p*_min_ = 426.9 ± 17.9 MPa	*p*_1_ = 424.4 ± 9.9 MPa	
						*p*_5_ = 560.4 MPa	*p*_5_ = 560.7 MPa	*p*_5_ = 565.7 MPa	
*L.monocytogenes*	BCRC 15354	Whole milk	ND	ND	25 °C, 5 min	*k* = 0.114 ± 0.007 MPa^−1^	*k* = 0.114 ± 0.007 MPa^−1^	*n* = 3.9 ± 0.8	Huang et al. [43]
						*p*_min_ = 275.1 ± 6.9 MPa	*p*_min_ = 275.2 ± 7.3 MPa	*p*_1_ = 257.8 ± 24.5 MPa	
						*p*_5_ = 376.1 MPa	*p*_5_ = 376.2 MPa	*p*_5_ = 389.5 MPa	
					25 °C, 10 min	*k* = 0.099 ± 0.009 MPa^−1^	*k* = 0.100 ± 0.010 MPa^−1^	*n* = 2.9 ± 0.8	
						*p*_min_ = 237.5 ± 11.3 MPa	*p*_min_ = 237.7 ± 13.4 MPa	*p*_1_ = 208.4 ± 33.0 MPa	
						*p*_5_ = 353.8 MPa	*p*_5_ = 352.8 MPa	*p*_5_ = 363.0 MPa	
*Salmonella enteritidis*	Cocktail of 4 strains	Potato omelet	1.25 MPa·s^−1^	<1 s	21 °C, 5 min	*k* = 0.023 ± 0.002 MPa^−1^	*k* = 0.024 ± 0.002 MPa^−1^	*n* = 2.1 ± 0.2	Toledo et al. [44]
						*p*_min_ = 245.3 ± 12.7 MPa	*p*_min_ = 256.6 ± 19.0 MPa	*p*_1_ = 325.7 ± 23.4 MPa	
						*p*_5_ = 745.8 MPa	*p*_5_ = 736.3 MPa	*p*_5_ = 700.9 MPa	
*S. typhimurium*	DT 104	Whole milk	6.7 MPa·s^−1^	<10 s	21 °C, 10 min	*k* = 0.050 ± 0.006 MPa^−1^	*k* = 0.050 ± 0.007 MPa^−1^	*n* = 3.1 ± 0.8	Guan et al. [7]
						*p*_min_ = 334.6 ± 19.4 MPa	*p*_min_ = 337.4 ± 23.5 MPa	*p*_1_ = 339.9 ± 40.8 MPa	
						*p*_5_ = 564.9 MPa	*p*_5_ = 567.7 MPa	*p*_5_ = 571.3 MPa	
*Staphylococcus aureus*	Cocktail of 3 strains	Rice pudding	1.25 MPa·s^−1^	<1 s	23–27 °C, 10 min	*k* = 0.053 ± 0.007 MPa^−1^	*k* = 0.058 ± 0.008MPa^−1^	*n* = 3.2 ± 0.1	Pulido et al. [45]
						*p*_min_ = 284.5 ± 24.6 MPa	*p*_min_ = 305.8 ± 28.7 MPa	*p*_1_ = 314.0 ± 7.5 MPa	
						*p*_5_ = 501.7 MPa	*p*_5_ = 504.3 MPa	*p*_5_ = 519.2 MPa	
Total aerobic bacteria		Mango pulp	2 MPa·s^−1^	200 MPa·s^−1^	ND, 1 min	*k* = 0.032 ± 0.012 MPa^−1^	*k* = 0.033 ± 0.012 MPa^−1^	*n* = 2.6 ± 0.3	Lui et al. [46]
						*p*_min_ = 269.0 ± 8.2 MPa	*p*_min_ = 279.7 ± 7.8 MPa	*p*_1_ = 327.8 ± 21.5 MPa	
						*p*_5_ = 628.8 MPa	*p*_5_ = 628.6 MPa	*p*_5_ = 608.8 MPa	

^a^ CR, Compression rate; CT, Compression time. ^b^ DR, Decompression rate; DT, Decompression time. ^c^ The temperature given is either the initial or the process temperature of the treatment. ^d^ ND, Not determined.
